# Diagnosis and management of post-COVID (Long COVID) in children: a moving target

**DOI:** 10.1097/MOP.0000000000001221

**Published:** 2023-01-23

**Authors:** Rosa Morello, Laura Martino, Danilo Buonsenso

**Affiliations:** a Department of Woman and Child Health and Public Health, Fondazione Policlinico Universitario A. Gemelli IRCCS; b Centro di Salute Globale, Università Cattolica del Sacro Cuore, Rome, Italy

**Keywords:** children, Long coronavirus disease, postcoronavirus disease condition, SARS-CoV-2

## Abstract

This review describes recent findings about post-COVID condition (PCC, or Long COVID) in children, including current knowledge about its epidemiology, clinical presentation, pathogenesis and care.

**Recent findings:**

There is no internationally agreed definition of PCC, although now most researchers agree that it is a complex clinical symptomatology persisting for at least 3 months after COVID-19, without an alternative diagnosis. There are several uncertainties about paediatric PCC. So far, available literature suggest that 1–3% of recognized children with Severe Acute Respiratory Syndrome COronaVirus 2 (SARS-CoV-2) infection may develop PCC. Its pathogenesis is unknown, although there is increasing evidence about possible abnormalities in the immune responses, cellular metabolism and intestinal microbiota, along with chronic endothelitis.

**Summary:**

Management of PCC in children is complex and require a multidisciplinary approach, with the goal of offering the best care possible to support diagnostics, research, mental health and access to research projects.

## INTRODUCTION

The Severe Acute Respiratory Syndrome COronaVirus 2 (SARS-CoV-2) has posed several challenges to the global population, in terms of morbidity and mortality, economic impact and social implications. The clinical impact on children has been overall milder compared with adults, in terms of disease severity and deaths [1], although also in the paediatric population hospitalizations, deaths and severe acute complications like the Multisystem Inflammatory Syndrome (MIS-C) have been recorded globally [2]. Later during the pandemic, patients and early follow-up data highlighted that a group of adults were still reporting a number of persisting symptoms (e.g. fatigue, pains, malaise, gastrointestinal problems, neurocognitive problems, etc.) for months after initial infection [3]. This condition, known as Long coronavirus disease (COVID) or post-COVID condition (PCC), has been confirmed by several studies globally and, later, reported in the paediatric population as well by both healthcare professionals and parents’ organizations [4,5,6^▪^,7^▪^] In the awareness that PCC is a relatively recent medical entity and discoveries in the field are evolving fast, this article not only attempts to revise current knowledge of PCC in children focusing on epidemiologic and clinical aspects, but also tries to summarize current main pathophysiological hypotheses and potential approaches for patients’ care. This review will not consider MIS-C or other well characterized postacute consequences of COVID-19 (e.g. myocarditis, encephalitis or characterized neuroinflammatory conditions) [8–10] as PCC/Long COVID (despite being well characterized postacute consequences of SARS-CoV-2), but will focus on the definition provided below. 

**Box 1:**
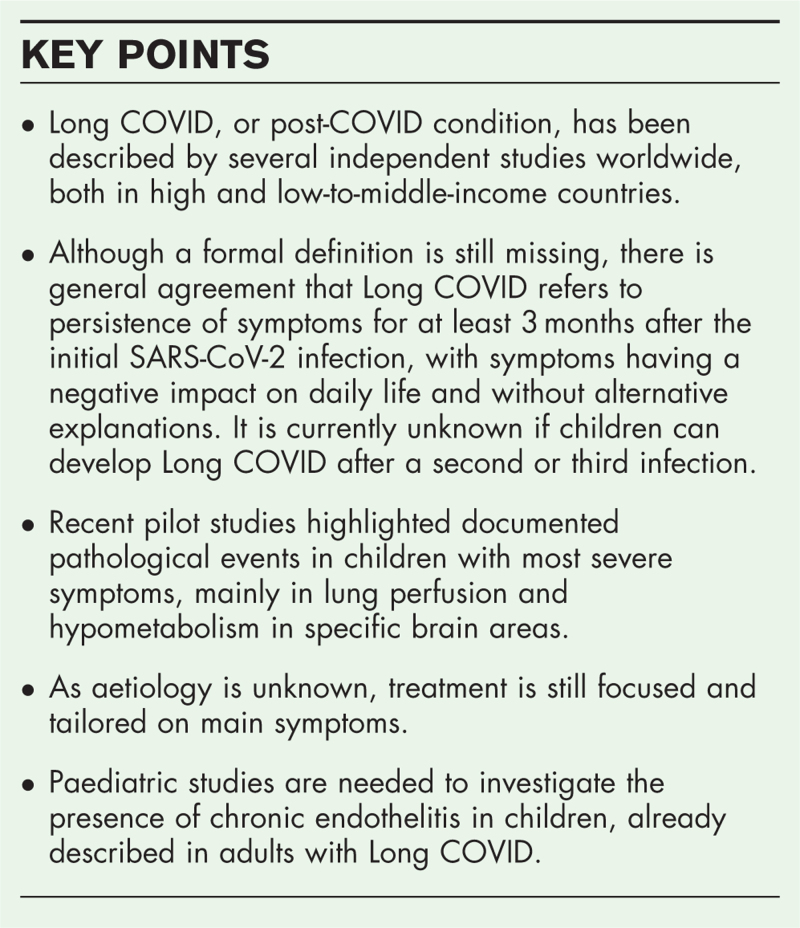
no caption available

## DEFINITION

To date (December 2022), there is no internationally recognized definition of PCC.

The WHO has released a definition of PCC in adults on October 2021: ‘PCC occurs in individuals with a history of probable or confirmed SARS-CoV-2 infection, usually 3 months from the onset of COVID-19 with symptoms that last for at least 2 months and cannot be explained by an alternative diagnosis. Common symptoms include fatigue, shortness of breath, cognitive dysfunction but also others which generally have an impact on everyday functioning. Symptoms may be new onset, following initial recovery from an acute COVID19 episode, or persist from the initial illness. Symptoms may also fluctuate or relapse over time’ (https://www.who.int/docs/default-source/coronaviruse/corrigendum-2021.1-postcovid-19-clinical-case-definition-2021-10-06-corr-2021-10-06-en.pdf?sfvrsn=1ebb697c_5). In this definition, the WHO highlighted that ‘A separate definition may be applicable for children’.

In the attempt to propose a specific definition for children, a multidisciplinary team from United Kingdom of the Clock study developed a DELPHI process, which finalized the following paediatric PCC definition, aligned to the WHO one: ‘PCC occurs in young people with a history of confirmed SARS-CoV-2 infection, with at least one persisting physical symptom for a minimum duration of 12 weeks after initial testing that cannot be explained by an alternative diagnosis. The symptoms have an impact on everyday functioning, may continue or develop after COVID infection, and may fluctuate or relapse over time. The positive COVID-19 test referred to in this definition can be a lateral flow antigen test, a PCR test or an antibody test’ [11^▪^]. Both the WHO and Clock definition highlight the importance of excluding other disorders that may present with similar signs and symptoms.

Currently, the WHO has also started a global consultation process, involving experts and family members, to provide a paediatric definition.

Despite the lack of definitive definition, currently most research and clinical programs are referring to the Clock definition [11^▪^], including this review. Importantly, it is still unknown if children can develop Long COVID after a second or third infection, as no studies investigating this have been published.

## EPIDEMIOLOGY

The real burden of paediatric PCC is still unknown and highly debated [6^▪^,^12^–^14^]. The lack of a standardized and internationally recognized clinical definition of PCC is the main barrier in understanding the epidemiology of PCC. For example, available studies so far have focused on different follow-up lengths for symptom persistence (from 1 to more than 3 months), or as considered as PCC the persistence of any symptoms, or at least the presence of multiple symptoms (e.g. three or more).

Also, study designs have been largely different. Early reports described symptom persistence of children with previous SARS-CoV-2 infection (usually including children with microbiologically confirmed infection) without a control group, with symptoms reported by parents or children, usually using self-filled surveys or during online interviews. These early reports reported extremely variable and high rate of symptoms persistence, from 5 to 40% of children reporting at least one persistence symptoms for more than 1–3 months after initial infection [15–23]. However, early studies have been criticized given the lack of a control group, as symptoms reported are vague and nonspecific and may be related to several other organic or psychological problems.

For these reasons, new funded studies have mostly focused on including control groups, using either children with no serologic evidence of previous COVID-19 or children that never received a positive molecular diagnosis of COVID-19 [24–29] were widely reported by all children, irrespective from having had or not previous COVID-19, letting the authors hypothesize that a significant psychological impact of the pandemic may explain the majority of symptoms reported in PCC studies. However, even such controlled studies all reported that symptoms were always more frequent in the group of previous infected children, with a difference ranging from 1 to 5% difference. It is important to highlight that even these study designs have been criticized, as false-negative serologic responses in children have been documented months after initial infection, and negative molecular tests does not exclude false-negative results or previous or later infections.

More recently, results from a more objective design has been published [30^▪^]. Rao *et al.* used electronic health record (EHR) to identify symptoms, signs, and diagnoses that were significant enough to prompt health service use. Nine U.S. children's hospitals participated, including individuals younger than 21 years who underwent antigen or reverse transcriptase–polymerase chain reaction (RT-PCR) testing for SARS-CoV-2 between 1 March 2020, and 31 October 2021, and had at least one encounter in the 3 years before testing. They compared syndromic (symptoms such as fever, cough, fatigue, shortness of breath, chest pain, palpitations, chest tightness, headache, and altered smell and taste), systemic (conditions such as as multi-inflammatory syndrome, myocarditis, diabetes, and other autoimmune diseases), and medication related with PCC identified in the 28–179 days following the initial test date. Six lakh fifty-nine thousand and two hundred and eighty-six children were included [52.8% boys, mean (SD) age 8.1 (5.7) years)], and 59 893 (9.1%) tested positive for SARS-CoV-2. The most common syndromic, systemic, and medication features associated with PCC were loss of taste or smell [adjusted hazard ratio (aHR), 1.96; 95% CI 1.16–3.32], myocarditis (aHR, 3.10; 95% CI 1.94–4.96), and cough and cold preparations (aHR, 1.52; 95% CI 1.18–1.96), respectively. The burden of PCC was estimated at 3.7% (95% CI 3.2–4.2).

Although even Rao's study design is not perfect (e.g. may have missed poorly classified problems like postexertional malaise or brain fog, or those seeking private care), the 3.7% estimate is closer to those from controlled studies, and more realistic. Considering that, in general, the number of paediatric COVID-19 cases has been probably underestimated because of the high number of asymptomatic/paucy-symptomatic cases, it is realistic to speculate that 1–3.5% of infected children can develop PCC. Importantly, this estimate may variate according to the SARS-CoV-2 variant causing the initial infection, or according to previous immunity (e.g. by vaccination or immunity), as happening with MIS-C, whose risk has now significantly decreased [31]. Importantly, given the methodological differences, so far it is not possible to provide data about regional or country-specific or ethnicity-specific estimates, although all controlled studies from UK, US, Switzerland suggest the 1–3% higher rates of symptoms in children with previous SARS-CoV-2 infection. Overall, published estimates from North America, UK, and Europe ranges from 1 to 20%.

Despite the mentioned uncertainties, there is more agreement between studies that children older than 10 years of age and those with allergic diseases are at higher risk of developing PCC [19]. Also, two independent studies have documented that PCC is less frequent and less severe (in terms of duration and symptoms burden) than in adults [32,33].

## CLINICAL PRESENTATION

**FIGURE 1:**
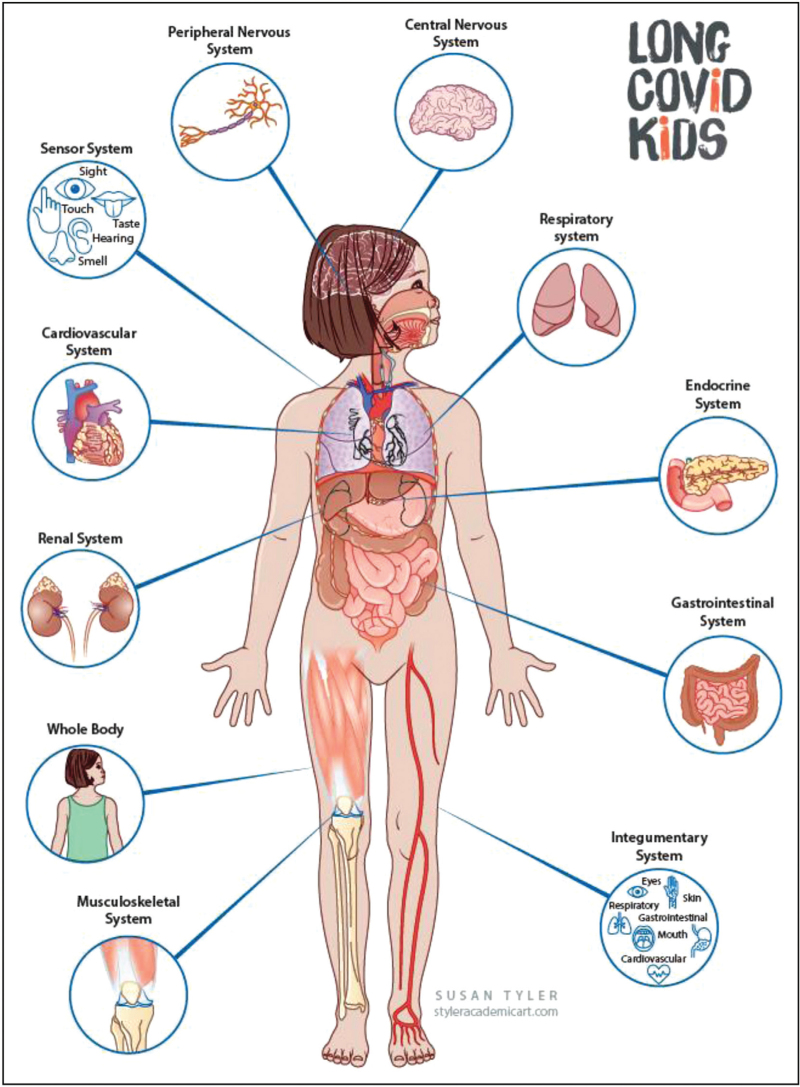
Patterns of persisting symptoms reported by published studies and family associations.

Despite the mentioned uncertainties related with the frequency of persistent symptoms reported by different studies, there is more agreement about the most frequent clusters complained by children with PCC or their parents. Several signs and symptoms have been reported by scientific studies or associations (Fig. 1) [6^▪^,^12^–30^▪^,^31^–^33^]. Fatigue is the most common symptom reported by all studies, in agreement with experiences from adult PCC clinics. Another frequent problems are postexertional malaise, even after mild activities, which usually does not allow the child to perform usual sports performed before COVID-19. Headache, gastrointestinal problems (nausea with or without vomiting, abdominal pain, or irregular stools), skin rashes, arthralgias and muscle pains, long-lasting alterations in smell and taste are all frequently reported. Several neurocognitive issues, including sleep problems, brain fog, changes in mood, tics, and so forth. All together, these symptoms have a negative impact on child's daily life: for either physical or cognitive problems, school attendance, return to sport and social activities are significantly impaired.

Overall, there is increasing agreement between experts (studies are still lacking) that PCC may not be a single disease but a cluster of different phenotypes of PCC (as proposed for Myalgic encephalomyelitis/chronic fatigue syndrome, ME/CFS) may be more realistic and appropriate for real-life settings. Also, such an approach may be more appropriate for clinical studies and pharmacological trials. For example, there are patients with prevalent neurocognitive problems, other with cardiopulmonary issues, or gastrointestinal problems, or different combinations of them but still with a cluster of more impactful symptoms.

As described, signs and symptoms complained by children with Long COVID are nonspecific and may be similar to those that characterize classic paediatric disorders, which, therefore, should be excluded in these children with chronic symptoms after SARS-CoV-2 infection. Fatigue can be because of anaemia, hypothyroidism or haematologic conditions, which should be excluded by cell blood count and thyroid function. Muscle and joint pains can be clues for rheumatologic disease and, therefore, screening for autoimmunity can be reasonable. Chronic abdominal pain with or without nausea, vomiting or diarrhoea can be clues of celiac disease, parasitic infections, liver or kidney issues that can be ruled out with specific testing.

## PATHOGENESIS

**FIGURE 2:**
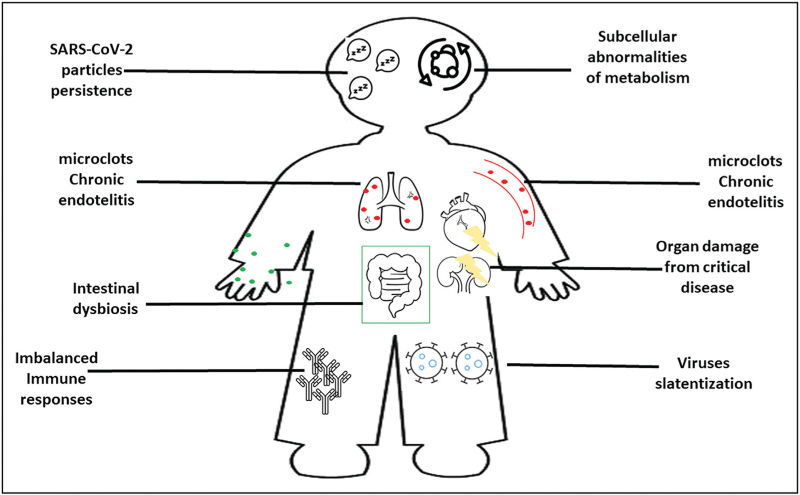
Summary of proposed postcoronavirus disease condition pathogenesis.

The pathogenesis of PCC is still unknown, although the number of published studies that have investigated possible biomarkers or events leading to PCC have significantly increased during the last year, particularly in adults. The most important aetiological hypothesis, currently supported by preliminary data and under further investigations, are summarized in Fig. 2 and detailed below: latency of SARS-CoV-2; reactivation of herpes viruses; chronically imbalance immune responses; intestinal dysbiosis; chronic endothelitis with circulating microclots; subcellular abnormalities in metabolic pathways; organ damage [34].

(1) Latency of SARS-CoV-2: there is overwhelming evidence that SARS-CoV-2 genome, at least parts of it, have been documented months after acute infection, initially in animal models and later confirmed in adults undergoing biopsies or postmortem autopsies [35]. Paediatric studies have documented similar studies: postmortem autopsis of children with MIS-C, which had previous mild or asymptomatic infection have documented widespread evidence of SARS-CoV-2 months after initial infection, including in the heart, intestine, and brain [36], and a multicentre study that performed intestinal lymph node biopsies of children with intussusception also documented SARS-CoV-2 particles [37]. SARS-CoV-2 has also been documented in the intestinal bowel months after infection in several studies, including two children [38,39]. The meaning of these findings are unknown, current hypotheses speculate that SARS-CoV-2 particles may stimulate subclinical chronic inflammation.
(2) Reactivation of herpes viruses: independent large studies in adults have documented reactivations of Epstein–Barr and other herpes viruses, speculating that the acute infection reactivate previously latentized viruses which, in turn, contribute to the development of chronic symptoms [40].
(3) Chronically imbalance immune responses: chronically aberrant immune signatures in adults with PCC have been widely documented, although the pathological events leading to these signatures in some patients and not others are still unknown [41]. Adult studies and patients organizations have also documented raised levels of autoimmunity markers, like ANA and ANCA [42]. Autoantibodies have also been documented [43]. In children, wide variability in Treg signatures have been documented with a subgroup of PCC children showing a Treg expression pattern more similar to children with acute infection than those that fully recovered, suggesting that possible chronic inflammation is ongoing [44^▪^], but larger studies are still needed.
(4) Intestinal dysbiosis: the evidence of SARS-CoV-2 persistence led to development of several researches assessing intestinal barrier in both PCC and MIS-C. In MIS-C, intestinal dysbiosis and abnormalities in the intestinal permeability have been documented [45]. It has been speculated that intestinal abnormalities can alter the gut–brain axis and contribute with chronic intestinal and neuropsychiatric symptoms [46].
(5) Chronic endotheliitis with circulating microclots: early during the pandemic, it has become evident that cardiovascular events (thrombohaemorragic events) were frequently complicated severe COVID-19 in adults. Also, a significant burden of similar events in the postacute phase of adults with COVID-19 have been clearly documented [47^▪^–49^▪^]. These observations led researchers to focus on markers of endothelial disorder as possible biomarkers of patients that never recovered from COVID-19. Abnormalities in coagulation markers have been documented in adults and, less severely, also in children [50^▪^]. Pretorius *et al.* have documented in different studies, including both PCC and ME/CFS patients, circulating microclots, a possible indirect marker of chronic endothelial/platelet activation. Although the role of these findings is still not established, the hypothesis is that these events may lead to abnormalities in the peripheral circulation and tissue oxygenation, contributing to symptoms like fatigue and PEM. In children, two studies have documented abnormalities in lung perfusion, which somehow may support such hypotheses [51^▪^,^52^^▪▪^].
(6) Subcellular abnormalities in metabolic pathways: several abnormalities have been documented in mitochondrial and oxidative pathways, which may contribute to the systemic and subclinical symptoms, which are difficult to be detected by routine diagnostics [53,54].
(7) Organ damage: severe organ damage from critical acute disease can explain persisting symptoms, although this seems more relevant in adults, as critical COVID is very rare in children. Importantly, in an immunological study of children with Long COVID, cytokine expression was not increased and did not differ from recovered children [44^▪^].

## DIAGNOSTICS

As the aetiology of PCC is unknown, biomarkers of this condition are not yet available. Therefore, a definitive guidance on how to approach diagnosis of PCC in children is still unavailable, although approaches used for similar clinical scenarios plus recent discoveries can help clinicians in developing local pathways. We have presented our clinical and diagnostic pathways more in details elsewhere [6^▪^], more recently guidances have been released by the American Academy of Pediatric and of Physical Medicine and Rehabilitation as well (https://www.aap.org/en/pages/2019-novel-coronavirus-covid-19-infections/clinical-guidance/postcovid-19-conditions-in-children-and-adolescents/) [55^▪^].

Briefly, all children with SARS-CoV-2 infection should undergo history and clinical examination at 1 and/or 3 months follow-up, to determine if they have persisting symptoms, which impact on daily life.

For children with persisting symptoms, a first step based on routine blood tests (blood cell count, liver and renal function, glucose, coagulation), electrocardiogram, lung ultrasound and exclusion of known and treatable diseases with similar presentation (e.g. hypothyroidism, autoimmunity, celiac disease) seems a reasonable approach. In adults, cardiopulmonary exercise testing is now seen as a gold standard to document fatigue or postexertional malaise (PEM), preliminary data on children are available [51^▪^]. As a noninvasive diagnostic, all centres might implement pathways including it in PCC children. Holter electrocardiogram (ECG) and tilt test may prove useful in children with Postural Orthostatic Tachycardia Syndrome (POTS-like) symptoms, while imaging can be reserved for specific scenarios (brain MRI in children with chronic headache, brain PET for severe cognitive problems).

With the growing evidence of chronic endothelitis and microclots in adults with PCC, inclusion of children as well in research settings testing them would be welcome. So far, indirect evidence of chronic endothelitis have been documented by two independent paediatric studies in Italy and Germany, using lung Single Photon Emission Computed Tomography (SPECT) or MRI, respectively [51^▪^,^52^^▪▪^].

The flow-chart we use in our centres has been presented elsewhere [6^▪^,51^▪^]. Briefly, children with persisting symptoms at 3 months perform initial routine tests to rule out other treatable conditions and, if all tests are normal, undergo a personalized second-level and third-level multidisciplinary diagnostics based on the child's main complaints. We are not aware of other guidance that suggest specific diagnostic pathways for children with PCC, as mostly only include general principles of care and exclusion of other conditions, with the exception of an extensive and well detailed guidance from the United States, which basically support a personalized strategy for diagnostics and care based on main complaints [55^▪^].

## CARE

Although there are no proven treatments for PCC in both adults and children, all children and families should be offered the best care possible, hopefully entering multidisciplinary pathways, which include the family paediatrician and different specialists in dedicated centres. Patients deserve to be offered the best care for their presentation irrespective whether their symptoms are related to COVID-19 or not.

A first essential step is to openly explain to families what is known and unknown about PCC, the importance of reciprocal trust, commitment in being updated with new discoveries and linked with other local, national or international teams dedicated to PCC, to share experiences and preliminary data.

In the best scenarios, all children should be included in clinical studies aimed at understanding paediatric PCC burden, length of illness, impact on social life, phenotypes and diagnostics. If possible, blood samples should be stored in biobanks or included in biomarker studies.

For children complaining symptoms that are frequently reported in paediatrics (e.g. chronic headache, abdominal pain, palpitation or POTS, sleep disturbances), clinicians should follow available guidelines, irrespective of the link with COVID-19 or not, as also mentioned in this US guidance [55^▪^]. For more complex scenarios, ideally children should be in clinical trials. Attractive options are nutraceuticals, lactoferrin, anticoagulation/aggregation [39].

As children, routine is usually disrupted in the context of PCC, certificates (or direct communication) for school members, sport tutors, or other close relatives and friends to allow personalized schedules or awareness from close people may be particularly useful, supporting the child in a gradual re-inclusion in the society and more support from close friends.

Irrespectively if PCC is an organic disease or a psychological condition, mental health support should always be guaranteed, when the child (and family) mental health looks compromised. As PCC children usually have a sudden change in their quality of life, psychological consequences are not infrequent and deserve attention and support, using the same approach advised for any child with mental health issues [55^▪^].

Importantly, many patients or experts are suggesting for nutraceuticals for the treatment of PCC in children [6^▪^]. So far, no studies have been published in children, and the most solid trial in adults did not find any differences in outcomes [56,57]. A case series from our team showed improvement of chronic gastrointestinal symptoms in children [39], but we are not having benefits for other systemic symptoms. Importantly, there is now no consensus for exercise therapy in children with PCC, as physical efforts can lead to worsening symptoms.

## PREVENTION

So far, therapeutic interventions that are able to prevent the development of PCC after SARS-CoV-2 infection are not known. Studies in adults have documented that COVID-19 vaccinations can significantly decrease the risk of PCC after breakthrough infection but not eliminate the risk [58]. Although paediatric evidence is not available on the role of vaccination, we have evidence that previous immunity can also prevent the development of MIS-C, and we can speculate that a similar effect on PCC development can be expected in children as well (while awaiting better evidence). Therefore, the only available strategy to prevent PCC is to avoid SARS-CoV-2 infection. However, it is important to know that nonpharmacological interventions implemented to prevent community spread of SARS-CoV-2 have had a devastating effect on child mental and physical health [59], therefore it is auspicable that families and societies implement balanced approaches.

## FUTURE PERSPECTIVES

Paediatric studies should aim to investigate at least:

(1) Biomarkers of PCC, including genomics, proteomics, autoantibodies, chronic endothelitis.
(2) Detailed clinical characterization of the different phenotypes of PCC (e.g. details of clinical signs and symptoms, long-term, results from different levels of diagnostics), which would be necessary to develop appropriate and focused inclusion criteria for treatment trials.
(3) Role of vaccination in preventing PCC in children.
(4) Randomized controlled trials about different pharmacological approaches.
(5) Multicentre multicountry studies would be highly necessary, although so far not yet planned. An International network of physicians from different expertise have been developed (the International Paediatric Post-COVID Condition in Children Collaboration), which organize virtual meetings every 2 months sharing updates, case discussions and new priorities [5]. Currently, results from an analysis of strategies implemented by different centres of this network is under review.

## CONCLUSION

There is increasing understanding of the impact of PCC in children. Although several questions are still unanswered, our knowledge and understanding is improving, providing hope for the future. In the meantime, clinicians should use the available knowledge to implement local PCC clinics and studies, and governments should understand the negative effects of PCC in children and fund appropriate diagnostics and therapeutic trials.

## Acknowledgements

*We are grateful to collaborators and family associations worldwide that collaborated with us during the last 2 years in better understanding and caring for paediatric Long COVID. We are especially grateful to LongCovidKids UK for providing us*Fig. 1.

### Financial support and sponsorship

*None.*

### Conflicts of interest

*D.B. has received grants to study Long COVID by Pfizer and Roche Italia.*

## REFERENCES AND RECOMMENDED READING

Papers of particular interest, published within the annual period of review, have been highlighted as:

▪ of special interest
▪▪ of outstanding interest
